# Scubcutaneous *Mycobacterium marinum* infection misdiagnosed as sporotrichosis: A case report

**DOI:** 10.1097/MD.0000000000032220

**Published:** 2022-12-09

**Authors:** Shi Chen, Zehu Liu, Xiujiao Xia

**Affiliations:** a Department of Clinical Laboratory; b Department of Dermatology, Hangzhou Third People^’^s Hospital, Affiliated Hangzhou Dermatology Hospital, Zhejiang University School of Medicine, Hangzhou, China.

**Keywords:** *Mycobacterium marinum*, sporotrichosis, tropical ornamental fish

## Abstract

Nontuberculous mycobacteria (NTM), are natural inhabitants of natural waters, engineered water systems, and soils. As a consequence of their ubiquitous distribution, humans are surrounded by these opportunistic pathogens.

**Patient concerns:**

In this report, we describe a case of scubcutaneous *Mycobacterium* marinum infection associated with home tropical ornamental fish aquaria. A 43-years-old man reported progressively increasing nodules over his left forearm and hand for more than 7 months.

**Diagnosis:**

Based on NTM culture, pathological examination, identification by gene sequencing and matrix assisted laser desorption ionization time of flight mass spectrometry, the diagnosis of scubcutaneous NTM infection was confirmed.

**Interventions:**

The patient was treated with itraconazole for suspected sporotrichosis over 1 month.

**Outcomes:**

The patient was treated with oral doxycycline hydrochloride capsules (200 mg/day) for 5 months, the nodules had resolved.

**Conclusion:**

Home aquarium environments may serve as a possible source of mycobacteria infection in urban family.

## 1. Introduction

Nontuberculous mycobacteria (NTM) are normal inhabitants of a variety of environmental habitats that are shared with humans and animals, including natural waters, drinking water distribution systems, and soils.^[1]^ Of these, *Mycobacterium marinum* is a slowly growing pigmented NTM responsible for “fish tank granuloma” due to its ability to cause localized skin and soft tissue infections in individuals who suffered puncture injuries or other types of trauma in freshwater or salt water.^[2]^

## 2. Case presentation

**Figure 1.**
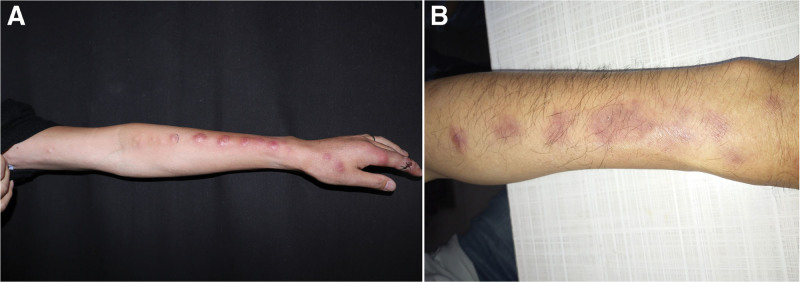
Sporotrichoid form of skin lesions (a), complete healing of the lesions following therapy (b).

**Figure 2.**
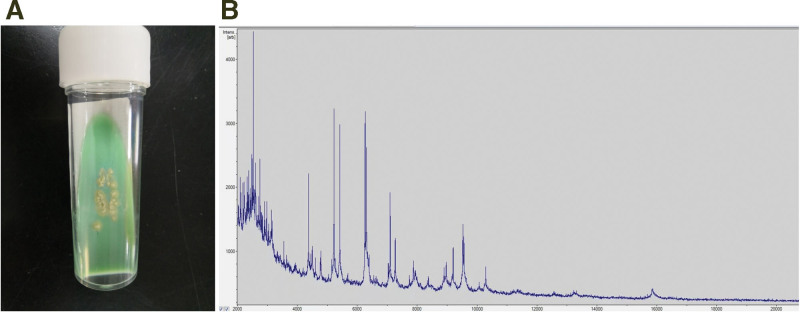
Subculture of colonies on Lowenstein-Jensen solid medium after 7 days of incubation at 25°C (a), Mass spectrum of the isolate of *M. marinum* with a score > 2.0 (b). *M. marinum* = *Mycobacterium marinum.*

A 43-years-old man presented to the dermatology clinic with a 7-month history of progressively increasing nodules over his left forearm and hand. The patient worked as a senior management of a company and owned a home aquaria with many tropical ornamental fish. The patient noted that he suffered an accidental injury to his finger while changing water for fish tank. He had been evaluated at another clinic, where he received treatment with itraconazole for suspected sporotrichosis. However, no improvement was seen after treatment of three months. Physical examination revealed sporotrichoid nodular lymphangitis of the left arm (Fig. 1a). A biopsy specimen was obtained and revealed granulomatous infiltrate with epithelioid and giant cells in hematoxylin-eosin staining. Tissue and exudates was inoculated onto multiple sets of Lowenstein-Jensen solid medium and were incubated at 25°C, respectively, and after 7 days growth, tiny and yellowish colonies were seen. The colonies were further transformed and purified, 7 days later, the yellow colonies was folded like chrysanthemum (Fig. 2a). A subculture of the isolate was submitted to Shanghai Sangong Biotech (Shanghai, China) for 16S ribosomal RNA gene sequencing, A basic local alignment search tool search of the GenBank database showed that the gene for 16S ribosomal RNA, partial sequence from the isolated pathogen (GenBank accession no. OM691430) had 100% homology with *Mycobacterium marinum* (*M. marinum*) (GenBank accession no. CP024190.1). Another subculture of the isolate was submitted to identification by matrix assisted laser desorption ionization time of flight mass spectrometry (Bruker Daltonik MALDI Biotyper). The pretreatment method of mass spectrometry was based on literature and manufacturer’s suggestions.^[3]^ Extended direct transfer sample preparation procedure was performed in the biosafety cabinet. The picked colony was smeared as a thin film directly onto a sample position of a 96-plate polished steel target plate using a sample applicator, 1 μl 70% formic acid was then added to overlay the sample position. 1 μl of Bacterial Test Standard (BTS) was deposited onto a assigned BTS quality control position of the steel target plate. After sample position and BTS quality control position were air-dried at room temperature, the positions were overlayed with 1 μl α-cyano-4-hydroxycinnamic acid (HCCA) matrix solution. When the spots were air-dried again, the prepared steel target plate was loaded into the mass spectrometer. Acquisition of the spectra was done in the range of a mass/charge (m/z) ratio of 2000 to 20,000. The composite profile was analyzed using the Bruker MALDI Biotyper software (version 3.1) with the standard reference Mycobacteria Library (version 4.0) database. Scoring was done as per criteria established by Bruker Daltonics. The spectral patterns maximum protein peak intensity > 1000 were compared with the Bruker Mycobacteria Library database for identification, the result was *M. marinum* with score of 2.007 (Fig. 2b). Susceptibility testing for 16 antibiotics as determined by agar dilution on the isolate was carried out. minimum inhibitory concentration distributions are given in Table 1. Finally, *M. marinum* infection was confirmed. The patient was treated with oral doxycycline hydrochloride capsules (200 mg/day) for 5 months, the nodules had resolved (Fig. 1b).

**Table 1:** Distributions of *MICs* of 16 antibiotics obtained by agar dilution against *M. marinum.*

Antibiotic	MIC (mg/L)	Interpretation
Meropenem	4	Susceptible
Trimethoprim/Sulfamethoxazole	9.5/0.5	Susceptible
*Linezolid*	2	Susceptible
*moxifloxacin*	1	Susceptible
*Ciprofloxacin*	1	Susceptible
Imipenem	≤2	Susceptible
Cefoxitin	64	Intermediary
Tygacil	1	Susceptible
*Tobramycin*	8	Resistant
Doxycycline	2	Intermediary
AmoxiciHin-clavldantic acid	>64/32	Resistant
Clarithromycin	≤1	Susceptible
Minocycline	2	Intermediary
Amikacin	≤2	Susceptible
Rifampicin	≤0.25	Susceptible
Rifabutin	≤0.5	Susceptible

*M. marinum* = *Mycobacterium marinum*, MIC = minimum inhibitory concentration.

### 2.1. Ethical approval and consent

Written and informed consent was obtained from this patient. The study was approved by the Medical Ethics Committee at the Department of Dermatology, Hangzhou Third People’s Hospital.

## 3. Discussion

The incidence of mycobacterial species in aquarium fish and water environments were as high as 42.9% and 75.4% respectively.^[4]^ So, aquarium environments may serve as a possible source of mycobacteria infection in urban. Clinically, it is difficult to distinguish cutaneous infection caused by *M. marinum* from sporotrichosis, in our study, the patient had already received (ineffective) antifungal agents due to misdiagnosis as sporotrichosis. Positive rates of NTM culture ranged from 2.9% to 96.8%.^[5]^ *M. marinum* has an optimal growth temperature of 25°C, colonies grown on Lowenstein-Jensen solid medium are wrinkled, and faint yellow after exposure to light. matrix assisted laser desorption ionization time of flight mass spectrometry technology can quickly and effectively identify NTM.

## Author contributions

**Conceptualization:** Xiujiao Xia.

**Data curation:** Shi Chen.

**Formal analysis:** Shi Chen.

**Funding acquisition:** Zehu Liu.

**Methodology:** Shi Chen.

**Project administration:** Xiujiao Xia.

**Writing – original draft:** Xiujiao Xia.

**Writing – review & editing:** Shi Chen, Zehu Liu, Xiujiao Xia.
